# Completion Rate and Positive Results Reporting Among Immunotherapy Trials in Breast Cancer, 2004-2023

**DOI:** 10.1001/jamanetworkopen.2024.23390

**Published:** 2024-07-19

**Authors:** Marco Mariani, Giulia Viale, Barbara Galbardi, Luca Licata, Carlo Bosi, Matteo Dugo, Giulia Notini, Matteo M. Naldini, Maurizio Callari, Carmen Criscitiello, Lajos Pusztai, Giampaolo Bianchini

**Affiliations:** 1 Università Vita-Salute San Raffaele, Milan, Italy; 2 Department of Medical Oncology, IRCCS Ospedale San Raffaele, Milan, Italy; 3 Fondazione Michelangelo, Milan, Italy; 4 Division of Early Drug Development, European Institute of Oncology, IRCCS, Milano, Italy; 5 Department of Oncology and Hemato-Oncology, University of Milano, Milano, Italy; 6 Department of Internal Medicine, Section of Medical Oncology, Yale School of Medicine, New Haven, Connecticut

## Abstract

**Importance:**

Clinical trials are the path to test and introduce new therapies in the clinic. Trials that are unable to produce results represent inefficiency in the system and may also undermine patient confidence in the new drug development process.

**Objectives:**

To survey the immunotherapy clinical trial landscape of breast cancer between January 2004 and April 2023 and examine what fraction of trials with primary completion date up to November 30, 2022, failed to report outcome, assessing the proportion of trials that yielded positive results and describing trial features associated with these 2 outcomes.

**Design, Setting, and Participants:**

This cross-sectional study included breast cancer immunotherapy trials identified in ClinicalTrials.gov. Trial details and results were retrieved in December 2023. Google Scholar, PubMed, and LARVOL CLIN websites were also searched for reports.

**Main Outcomes and Measures:**

Trial outcome reported as abstract or manuscript. Reported trials were categorized as positive (ie, met its end point) or negative. Association between reporting and trial features were tested using Fisher exact test.

**Results:**

A total of 331 immuno-oncology trials were initiated in breast cancer by April 2023; 242 trials were phase II, 47 were phase I, and 42 phase III. By setting, 212 studies (64.0%) were conducted in metastatic, 94 (28.4%) in neoadjuvant, and 25 (7.6%) in adjuvant settings. Among phase II and III trials, 168 (59.2%) were nonrandomized. One hundred twenty trials had primary completion dates up to November 30, 2022, of which 30 (25.0%; enrolling a combined 2428 patients) failed to report their outcomes; 7 phase I trials (31.8%), 21 phase II trials (23.6%), and 2 phase III trials (22.2%) were unreported. Single-center studies were significantly more likely to be unreported than multicenter studies (19 of 54 [35.2%] vs 9 of 60 [15.0%]; *P* = .02). Of the 90 reported trials, 47 (52.2%) and 43 (47.8%) were positive and negative, respectively. Seventeen of 19 (89.5%) of the reported randomized trials (accruing a total of 4189 patients) were negative.

**Conclusions and Relevance:**

In this cross-sectional study of immunotherapy breast cancer trials, the large number of trials yielded modest clinical impact. Single-center trials commonly failed to report their outcomes and many phase II studies have not translated into corresponding successful phase III trials.

## Key Points

**Question:**

What fraction of immunotherapy trials launched since 2004 have reported an outcome and translated into positive phase III randomized trials, and what features are associated with failure to report results?

**Findings:**

In this cross-sectional study of 331 immunotherapy breast cancer trials, 25% of the 120 trials with a primary completion date before December 2022 failed to report their outcomes (31.8%, 23.6%, and 22.2% of phase I, II, and III trials, respectively), and 89% of the 19 randomized trials reported negative results. Single-center studies were significantly more likely to be unreported.

**Meaning:**

Trials that are unable to produce results or fail to translate into successful phase III trials represent inefficiency in the clinical trial system and increase drug development costs; the results of this study suggest that the many single-center, small, unrandomized phase II trials appear to be low yield.

## Teaser

This cross-sectional study assesses the proportion of immunotherapy trials for breast cancer since 2004 that have failed to report their outcomes, the proportion of these trials that have yielded positive results, and the features associated with both outcomes.

## Introduction

The modern era of cancer immunotherapy started with the demonstration of the remarkable single-agent efficacy of ipilimumab in metastatic melanoma in 2010.^1^ Historically, breast cancer was considered not to be an immunogenic cancer.^2^ Nevertheless, extensive biomarker research has established the prognostic and chemotherapy response predictive value of immune infiltration in breast cancer,^3,4,5,6^ and preclinical studies have suggested that immune cell activation mediates chemotherapy effect and provide antitumor immune surveillance.^7^ These observations motivated launching many immunotherapy trials in the early 2010s in breast cancer that leveraged clinical experience with immune checkpoint inhibitors in other cancer types. However, as of December 2023, there is only 1 immunotherapy drug, pembrolizumab, that is approved for the treatment of breast cancer in the US under 2 distinct indications; as first-line therapy in combination with chemotherapy for programmed death ligand 1 (PD-L1)-positive (Combined Positive Score [CPS] score 10 or above) metastatic triple negative breast cancer (mTNBC),^8^ and as neoadjuvant therapy in combination with chemotherapy followed by adjuvant pembrolizumab for stage II/III TNBC.^9^ In Europe, atezolizumab is also available as first-line therapy in combinations with nab-paclitaxel for PD-L1–positive (Immune Cell [IC] score 1% or higher) mTNBC.^10^ This indicates surprisingly low societal and pharmaceutical industry return on the large number of trials conducted in this clinical space in the past 15 years. The goal of our analysis was to survey immunotherapy trials in breast cancer and assess what fraction of trials that met their prespecified completion time points have reported outcomes. We also examined what trial features were associated with failure to report results, and what fraction of completed and reported randomized trials met their primary end point.

## Methods

Breast cancer immunotherapy trials were identified in the ClinicalTrials.gov website in April 2023 using the search terms *pembrolizumab, camrelizumab, nivolumab, toripalimab, sintilimab, tislelizumab, cemiplimab, spartalizumab, dostarlimab, pucotenlimab, balstilimab, carilizumab, retifanlimab, serplulimab, atezolizumab, durvalumab, avelumab, TQB2450, pacmilimab, erfonrilimab, *and *envafolimab* appearing with *cancer vaccine, CAR-T, immunomodulators, adoptive T-cell therapy, *and *immunotherapy* (eFigure in Supplement 1). Trial features including sample size (categorized by number of patients enrolled into trials enrolling fewer than 50 patients, 50 to 200 patients, and over 200 patients), design (randomized or nonrandomized), trial phase (I, II [including phase I/II studies], III [including phase II/III]), disease setting (neoadjuvant, adjuvant, or metastatic), site (single-center [defined as trial conducted at only 1 institution] or multicenter), primary end point, lead sponsor (industry, National Institute of Health [NIH], others), primary completion date, and results were extracted for each trial in December 2023. The database is available as an Excel table (eTable in Supplement 2). According to Food and Drug Administration (FDA) legislation, trials are required to report results in ClinicalTrials.gov within 1 year of primary completion date that is defined as “the date on which the last participant was examined or received an intervention to collect final data for the primary outcome measure, this term refers to the date on which data collection is completed for all the primary outcome measures.” We therefore restricted our reporting analysis to trials with primary study completion dates up to November 30, 2022. Outcome reports for these studies were retrieved from the ClinicalTrials.gov website and, via the National Clinical Trial (NCT) number, from Google Scholar, PubMed, and LARVOL CLIN.^11^ A trial was considered reported if results were posted on ClinicalTrial.gov or reported as an abstract or manuscript. Four trials never started accrual (NCT03554109, NCT03872505, NCT04088032, NCT04249167) and were excluded from the reporting analysis. We categorized trials that reported outcome as positive or negative based on whether the study met its primary end point. We then focused on randomized trials that reported outcome and excluded from this analysis 5 randomized trials (NCT02622074, NCT03167619, NCT03487666, NCT03566485, NCT04215146) that had noncomparative designs or had end points other than efficacy (eg, dose finding).

A single author (M. M.) performed the data abstraction. Although we attempted to use a standardized method for data extraction, we soon recognized its failure to capture the entirety of the reported study status and the quality of the study. Therefore, as described previously, we decided to manually filter, check the reporting status, and extract the results of each trial.

This was an analysis of publicly available aggregate trial data; thus, institutional review board approval and informed consent was not required per the Common Rule. This study followed the Strengthening the Reporting of Observational Studies in Epidemiology (STROBE) reporting guideline.

### Statistical Analysis

Associations between trial reporting outcomes and trial features of interest were tested using Fisher exact test. A 2-sided *P* < .05 was considered to be statistically significant, without correction for multiple testing. Analyses were performed with R software version 4.2.1 (R Project for Statistical Computing) package ggplot2.

## Results

**Figure 1.**
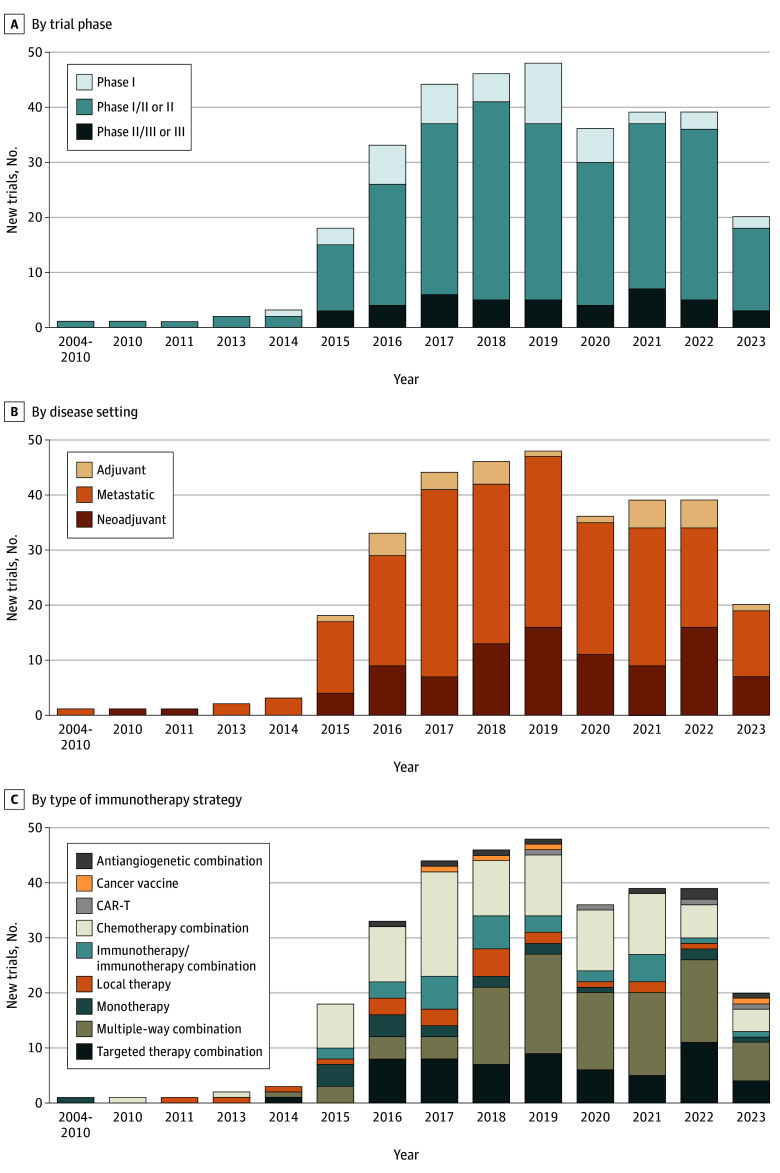
Overview of Immuno-Oncology Trials in Breast Cancer Opened Between 2004 and April 2023 Multiple way combination describes immunotherapy combined with several drugs with different mechanisms of action.

**Figure 2.**
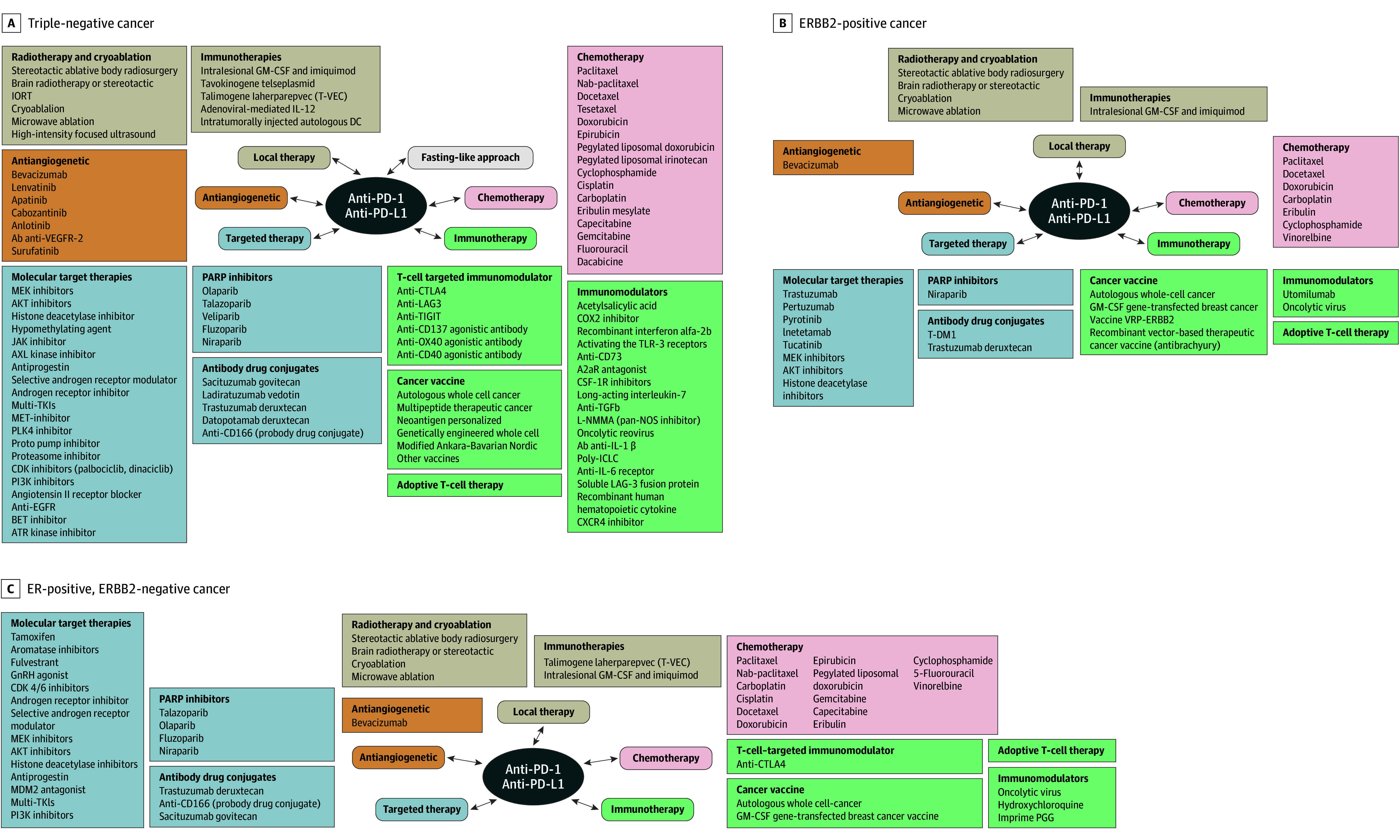
Landscape of Anti-PD-1 and Anti-PD-L1 Combination Trials in Breast Cancers CSF-1R indicates colony-stimulating factor-1 receptor; EGFR, epidermal growth factor receptor; GM-CSF, granulocyte-macrophage colony-stimulating factor; GnRH, gonadotropin hormone-releasing hormone; IL, interleukin; IORT, intraoperative radiation therapy; PARP, poly (adenosine diphosphate–ribose) polymerase; PD-1, programmed cell death protein 1; PD-L1, programmed death ligand 1; T-DM1, trastuzumab emtansine; TGFb, transforming growth factor-β; TKI, tyrosine kinase inhibitor; VEGFR-2, vascular endothelial growth factor; VRP, virus replicon particle.

**Figure 3.**
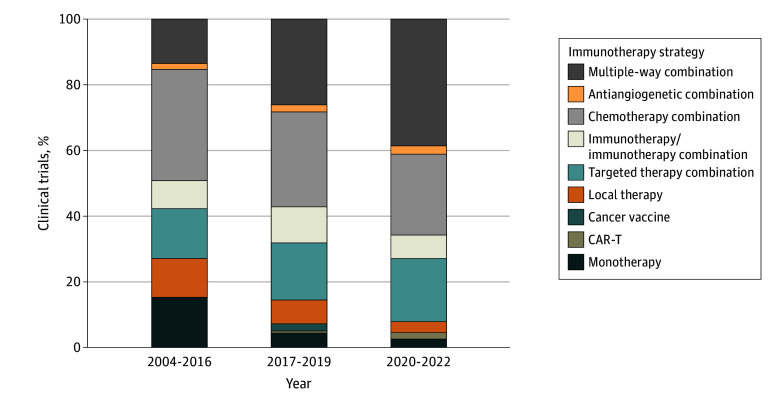
Evolution of Types of Immunotherapy Trials in Breast Cancer Updated as of December 31, 2022. Multiple way combination describes immunotherapy combined with several drugs with different mechanisms of action.

Three hundred and thirty-one immunotherapy trials were launched between January 2004 and April 2023, targeting to accrue 48 844 patients. Overall, 47 (14.2%), 242 (73.1%), and 42 trials (12.7%) were phase I, II, or III, respectively. Ninety-four (28.5%), 25 (7.5%), and 212 trials (64%) were conducted in the neoadjuvant, adjuvant, and metastatic disease settings, respectively. Two hundred and fifty-two trials (76.1%) were designed to include the TNBC subtype, while 79 (23.9%) included exclusively ER-positive and/or ERBB2 (formerly HER2)-positive patients. Overall, there were 8 NIH-sponsored trials (2.4%), 80 industry-sponsored trials (24.2%), and 243 trials (73.4%) sponsored by other funding sources. Most trials are combination studies with chemotherapy, immunotherapy, or targeted therapies, (Figure 1, Figure 2, and Figure 3). Among 284 phase II and III trials, 168 were nonrandomized.

At the time of the analysis, 207 trials had not yet met their primary completion dates, including 84 randomized trials, while 120 trials that enrolled 10 830 patients had primary completion date up to November 30, 2022. Thirty (25%) of these had not reported results; 7 phase I trials (31.8%), 21 phase II trials (23.6%), and 2 phase III trials (22.2%) were unreported. Out of the 30 unreported trials, 4 listed a terminated status. These unreported trials enrolled 2428 patients combined. Single-center studies were significantly more likely to be unreported than multicenter studies (19 of 54 [35.2%] vs 9 of 60 [15.0%]; *P* = .02) (Table 1). Among unreported single-center studies, 8 trials (42%) were conducted in America, 9 (47%) in Asia, 1 (5%) in Australia, and 1 (5%) in Europe. Despite FDA regulations, only 74 trials (61.7%) reported results in ClinicalTrials.gov, with additional 16 presenting results in the published research.

**Table 1.** Trial Features Associated With Not Reporting Results for Trials That Had Primary Completion Dates Before December 2022

Characteristic	Trials, No. (%)		*P* value^a^
	Reported (n = 90)	Unreported (n = 30)	
Sample size, No.			
<50	57 (63.3)	18 (60)	.95
50-200	22 (24.4)	8 (26.7)	
>200	11 (12.2)	4 (13.3)	
Allocation			
Randomized	24 (26.7)	10 (33.3)	.49
Nonrandomized	66 (73.3)	20 (66.7)	
Phases			
I	15 (16.7)	7 (23.3)	.72
I-II or II	68 (75.6)	21 (70)	
II-III or III	7 (7.8)	2 (6.7)	
Setting			
Neoadjuvant	21 (23.3)	9 (30)	.55
Adjuvant	2 (2.2)	1 (3.3)	
Metastatic	67 (74.4)	20 (66.7)	
Lead sponsor			
Industry	28 (31.1)	8 (26.7)	.86
NIH	1 (1.1)	0	
Other	61 (67.8)	22 (73.3)	
Center			
Single-center	35 (38.9)	19 (63.3)	.02
Multicenter	51 (56.7)	9 (30)	
NA	4 (4.4)	2 (6.7)	

Abbreviations: NIH, National Institutes of Health; NA, not available.

^a^ *P* values were calculated with Fisher test comparing reported vs unreported trials.

Overall, 47 of 90 trials (52.2%) were positive, and 43 of 90 (47.8%) were negative. When considering only the reported phase II trials, 31 of 68 (45.6%) were positive, and 37 of 68 (54.4%) were negative. Furthermore, among the positive trials, 26 of 47 (55.3%) were reported in a manuscript, whereas 32 of 43 (74.4%) of the negative trials were reported in a manuscript.

Of the 19 randomized trials that reported outcome excluding 5 randomized trials that had noncomparative designs or had end points other than efficacy (as described in Methods section), 17 (89.5%) had negative results, including 4 of the 6 randomized phase III trials (Table 2). The negative randomized trials combined enrolled 4189 patients. Due to the small number of positive trials (2 of 19 trials), no statistically significant association was found between trial features and positive vs negative results (Table 2).

**Table 2.** Trial Features Associated With Positive vs Negative Results in Randomized Trials That Had Primary Completion Dates Before December 2022

Characteristic	Trials, No. (%)		*P* value^a^
	Positive (n = 2)	Negative (n = 17)	
Sample size, No.			
<50	0	2 (11.8)	.57
50-200	0	8 (47.1)	
>200	2 (100)	7 (42.2)	
Phases			
I-II or II	0	13 (76.5)	.08
II-III or III	2 (100)	4 (23.5)	
Setting			
Neoadjuvant	1 (50)	3 (17.7)	.46
Adjuvant	0	1 (5.9)	
Metastatic	1 (50)	13 (76.5)	
Primary end point^b^			
pCR	1 (25)	3 (12)	.67
ORR	0	4 (16)	
PFS or EFS or IDFS	1 (25)	10 (40)	
OS	1 (25)	2 (8)	
Others^c^	1 (25)	6 (24)	
Lead sponsor			
Industry	2 (100)	11 (64.7)	>.99
NIH	0	0	
Other	0	6 (35.3)	
Center			
Single-center	0	2 (11.8)	>.99
Multicenter	2 (100)	14 (82.3)	
NA	0	1 (5.9)	

Abbreviations: EFS, event-free survival; IDFS, invasive disease-free survival; NIH, National Institutes of Health; ORR, overall response rate; OS, overall survival; pCR, pathological complete response; PFS, progression-free survival.

^a^ *P* values were calculated with Fisher test.

^b^ Co-primary end points are counted separately.

^c^ Others include safety, tumor-infiltrating lymphocytes increase, clinical benefit rate, and circulating tumor DNA clearance.

## Discussion

A total of 46 844 patients with breast cancer participated in 331 immunotherapy trials in the past 20 years, leading to a single drug approval under 2 separate indications in the US. Two hundred and seven trials have not yet met their primary completion dates, including 84 randomized trials, and new approvals will likely occur in the coming years. However, 120 trials had primary completion dates before December 2022 and 30 of these have not posted or published results. This is consistent with earlier studies that reported only around 40% 1-year reporting compliance across all trials, as well as oncology trials, in ClinicalTrials.gov; sponsors running many trials (eg, industry) were significantly more likely to be compliant than smaller sponsors.^12,13^ In our breast cancer focused immunotherapy trials analysis, we also found that single-center trials (typically smaller phase I/II trials) were significantly less likely to report results than multicenter studies.

Our examination of breast cancer immunotherapy trials across all phases revealed a numerically higher number (47) of positive trials compared with negative (43) ones. However, for some phase I and II trials reported only as abstracts, the statistical criteria for success were not provided. Consequently, these trials were considered positive if they reported tolerability and numerical efficacy results within the range of expected benefit rates from existing therapies in the given disease setting.

In clinical research, a significant hurdle is publication bias, resulting in greater prominence for studies with positive results, which are more likely to be published in high-impact journals and consequently cited more frequently.^14,15,16^ In this cross-sectional study of immunotherapy trials for breast cancer, it is noteworthy that there were numerically more negative trials reported as manuscripts (74%) compared with positive ones (55%). This could be due to the relatively early reporting of the results, and perhaps the manuscripts are still in preparation. However, Krzyzanowska et al^17^ reported that within 5 years after presentation at the meeting, 26% of randomized phase 3 oncology trials remain unpublished. Additionally, breast cancer had the highest unpublished rate at 36%, while lung cancer the lowest at 16%. The reasons behind this phenomenon are known and may affect all levels of the publication system, from the authors to the journals, and represents a break in what may be described as an implicit contract between investigators and trial participants that exerts a negative impact on the research community.^18^ High-quality research, acknowledgment of conflicts of interest, and publishing trial results regardless of their outcomes are all established strategies to minimize publication bias.^19^

Seventy-three percent of the trials conducted since 2004 were phase II studies (242 trials); results from these trials are supposed to guide phase III trial design to maximize the chance for positive outcome. Disappointingly, 89.5% of the completed randomized immunotherapy trials yielded negative results. The poor ability of phase II results to predict success in phase III trials has been known for several decades, and is attributed to patient selection, limited value of historical controls when judging efficacy, unreliable phase II end points (ie, progression-free survival), and compromises in phase II trial sample size and design to ensure feasibility in small academic settings and to control costs.^20,21,22^ Recognizing that these same issues continue to plague modern immunotherapy trials, the Society for Immunotherapy of Cancer (SITC) recently published a detailed framework to maximize the value and success of phase III trials in immuno-oncology.^23^ Many currently tested drug combinations are empirical and development of more human-relevant preclinical immunotherapy models are needed to generate better rationale for combination therapies and define the clinical niche where these might shine.^24^ Even the best preclinical models are unlikely to capture the variability in human antitumor immunity and therefore early identification of the treatment sensitive subpopulation through biomarker discovery is essential to increase the chance of success in phase III trials.^21,25^ Observing efficacy in patient populations that progressed on, or not sensitive to, current immunotherapy modalities also bodes well for future success in larger trials.^26^

### Limitations

We acknowledge several limitations in our study that are important for properly interpreting our findings. First, the data extraction from ClinicalTrials.gov was performed manually, which involved a thorough review and curation of each trial’s reported outcomes. While this approach allowed for detailed scrutiny of results, it inherently carries the risk of inaccuracies and potential biases in trial results interpretation to define positive or negative studies.

Second, our reliance on ClinicalTrials.gov as the primary source of trial data might have resulted in some trials being overlooked. ClinicalTrials.gov is a comprehensive registry, but it is not exhaustive. Other registries and sources could host information on trials that were not captured in our database, which might affect the generalizability of our findings.

Third, our analysis was restricted to trials with primary study completion dates before December 2022. This cutoff may exclude significant data from trials that concluded around or after this date but have not yet reported results. This time limitation might lead to an underrepresentation of more recent trials, which could influence the observed trends and conclusions regarding the effectiveness and reporting rates of immunotherapy trials in breast cancer.

## Conclusions

The findings of this study suggest that the large number of immunotherapy trials being run have yielded modest clinical impact. Single-center studies commonly fail to report outcome, and the many phase II studies that have been conducted have not translated into many successful phase III trials. More selective initiation of phase II trials, grounded in preclinical and biomarker observations and with optimal statistical designs for early efficacy assessment, is needed to increase trial efficiency.

## Notes

Supplement 1. **eFigure.** Flowchart of Methods

Supplement 2. **eTable.** List of Included Trials

Supplement 3.
Data Sharing Statement
