# Supplementary material for: Completion Rate and Positive Results Reporting Among Immunotherapy Trials in Breast Cancer, 2004-2023
Source: JAMA Netw Open. 2024 Jul 19;7(7):e2423390. doi: 10.1001/jamanetworkopen.2024.23390 (PMC11259908; doi:10.1001/jamanetworkopen.2024.23390)
Supplement: Supplement 1. — eFigure. Flowchart of Methods [file jamanetwopen-e2423390-s001.pdf]

Supplemental Online Content

Mariani M, Viale G, Galbardi B, et al. Completion rate and positive results reporting among immunotherapy trials in breast cancer, 2004-2023. *JAMA Netw Open.* 2024;7(7):e2423390. doi:10.1001/jamanetworkopen.2024.23390

Supplement 1. eFigure. Flowchart of Methods

This supplemental material has been provided by the authors to give readers additional information about their work.

© 2024 Mariani M et al. *JAMA Network Open*.

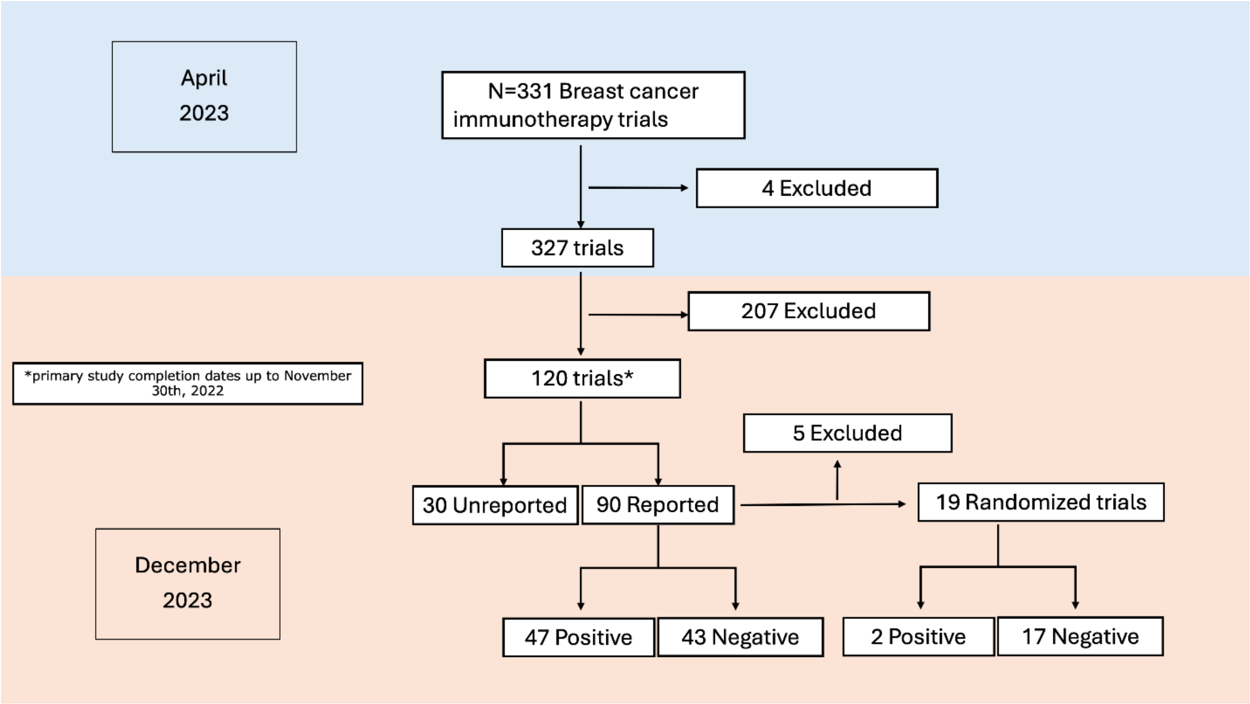

eFigure. Flowchart of Methods

© 2024 Mariani M et al. *JAMA Network Open*.
