# Supplementary material for: Completion Rate and Positive Results Reporting Among Immunotherapy Trials in Breast Cancer, 2004-2023
Source: JAMA Netw Open. 2024 Jul 19;7(7):e2423390. doi: 10.1001/jamanetworkopen.2024.23390 (PMC11259908; doi:10.1001/jamanetworkopen.2024.23390)
Supplement: Supplement 3. — Data Sharing Statement [file jamanetwopen-e2423390-s003.pdf]

Data Sharing Statement

Mariani. Completion Rate and Positive Results Reporting Among Immunotherapy Trials in Breast Cancer, 2004-2023. *JAMA Netw Open*. Published July 19, 2024.
doi:10.1001/jamanetworkopen.2024.23390

Data
Data available: Yes
Data types: Other (please specify)
Additional Information: publicly available data from clinicaltrial.gov
How to access data: https://clinicaltrials.gov
When available: With publication

Supporting Documents
Document types: Other (please specify)
Additional Information: excel file
How to access documents: excel file
When available: With publication

Additional Information
Who can access the data: anyone
Types of analyses: any purpose
Mechanisms of data availability: publicly available
